# Managing the Number of Tag Bits Transmitted in a Bit-Tracking RFID Collision Resolution Protocol

**DOI:** 10.3390/s140101010

**Published:** 2014-01-08

**Authors:** Hugo Landaluce, Asier Perallos, Ignacio Angulo

**Affiliations:** Deusto Institute of Technology (DeustoTech), University of Deusto, Bilbao 48007, Spain; E-Mail: ignacio.angulo@deusto.es

**Keywords:** RFID, anti-collision, tree, CT, window

## Abstract

Radio Frequency Identification (RFID) technology faces the problem of message collisions. The coexistence of tags sharing the communication channel degrades bandwidth, and increases the number of bits transmitted. The window methodology, which controls the number of bits transmitted by the tags, is applied to the collision tree (CT) protocol to solve the tag collision problem. The combination of this methodology with the bit-tracking technology, used in CT, improves the performance of the window and produces a new protocol which decreases the number of bits transmitted. The aim of this paper is to show how the CT bit-tracking protocol is influenced by the proposed window, and how the performance of the novel protocol improves under different conditions of the scenario. Therefore, we have performed a fair comparison of the CT protocol, which uses bit-tracking to identify the first collided bit, and the new proposed protocol with the window methodology. Simulations results show that the proposed window positively decreases the total number of bits that are transmitted by the tags, and outperforms the CT protocol latency in slow tag data rate scenarios.

## 1. Introduction

Radio frequency identification (RFID) technology is used for auto identification (auto-ID) as a replacement of the barcodes. RFID can wirelessly read codes that were previously stored in small transponders/tags. These tags are attached to different objects for monitoring and tracking in an omnidirectional fashion. The main idea of RFID is to tag every object in the world, so that everything can be identified, creating tremendous benefits in a very different kind of applications like traceability of goods, baggage management, livestock tracking, and supply chain management [1,2]. Typically, an RFID system comprises [3]:

— One or more tags. These include an IC-chip, an antenna and are attached to the objects to count or identify. Tags can be active (battery operated) or passive (no battery). Because passive tags are activated using coupled power originated from the reader, the latter has a lower coverage.
— A reader/interrogator. This device is made up of an RF module, a control unit and one or more antennas. It offers a bidirectional communication between the tags and the reader.
— A data processing subsystem. Connected to the reader, allows for the storage and further processing of the data information of identified tags into a database.

Unlike barcodes, RFID does not require imminent handling, no line of sight is required between the reader and the object to be identified, and tags provide greater storage (64 bits, 96 bits and 128 bits). Active tags can be equipped with sensors and constitute Wireless Sensor Networks [4]. However, since passive tags are cheaper than active tags, they are becoming more common in applications such as tracking, controlling and traceability. In addition, RFID is becoming a prominent technology in supply chain management and industrial automation applications, since it perfectly evolves into the paradigm of ubiquitous computing [5]. This fact defines RFID as a unique technology that allows ubiquitous identification.

The coexistence of various tags sharing the communication channel leads to a unique problem known as the tag collision problem. When various tags send messages to a reader simultaneously, a cancellation of bits is produced and the resulting message is unreadable (collision). Collisions force the reader to retransmit tag IDs, which results in a loss of bandwidth, an increase of power consumption, and a large delay in the identification process [3]. To face this problem an anti-collision protocol is needed. In literature, several proposed protocols have been reported, and they can be classified in aloha based, tree based and hybrid protocols [6] Aloha-based protocols are considered probabilistic because tags use random numbers to respond [7–9]. These protocols suffer from the tag starvation problem, in which a tag may not be read in a reading cycle. Tree based protocols [10] are considered deterministic and provide simple tag designs, e.g., the Query Tree (QT) [11]. These protocols read all the tags in the interrogation zone on each cycle. Hybrid protocols [12,13] are designed to avoid the problems of aloha and tree based protocols at the expense of a complex reader and tag designs.

In a Wireless Sensor Network, nodes use batteries like active RFID tags. To preserve these battery lives it is very important to control the amount of data transmitted and that is the main reason why we aim to preserve low complexity tags. Although tree based protocols require simple circuit designs on the reader and the tag side, they suffer from a great number of collisions. This cause the increase on the power consumption and the number of bits transmitted. Once tags have started transmitting, cannot stop until the last ID bit is transmitted, even in a collision situation, because they cannot support transmitting and receiving simultaneously [14] and do not have a sense of what is happening. Thus, tags whose ID match the query received will send the remaining ID from the last query bit received to the last bit, regardless of the kind of slot occurred. A window methodology is developed to alleviate this problem and decrease the number of tag bits transmitted. Tags matching the query, respond a fixed number of bits (window) indicated by a parameter, which decreases the number of tag bits wasted on collisions and therefore, the number of bits transmitted per tag is decreased. This methodology reduces the number of bits transmitted per tag, but the number of collisions is still a problem to be solved. Therefore, there is still room to improve the performance of the protocol.

In literature review, a new technology called bit-tracking has recently been adopted. This allows the reader to detect the locations of collided bits in a collision slot [15–17]. Methods using this technology actually decrease the number of slots due to the complete elimination of idle slots and the decrease in the number of collision slots.

In this paper, we propose to use our previously developed window methodology, whose positive results were presented in [18,19] applied to the QT protocol, with the collision tree (CT) protocol [17], which uses bit-tracking technology. In this manner, not only is the number of bits transmitted per tag decreased, but also the number of collisions. The proposed window allows the CT protocol to perform in the same manner as a bit by bit algorithm for small window size (*ws*) values or similarly to the CT for large *ws* values close to the ID length (*k*). We present a number of simulations to analyze the influence of the window in the CT protocol. Since the architecture of the RFID receivers, the encoding methods and the modulation used can determine tags data rate, and usually it is lower than the reader data rate [9], we have proposed a simulation considering reader and tags data rates, to conclude how this parameter affects the proposed window.

Subsequently, the rest of the paper is organized as follows: Section 2 provides background information and related work on anti-collision protocols. Section 3 presents the window methodology. In Section 4 applies the window to the CT protocol. In Section 5 presents the results of the evaluation of the CT protocol with different *ws* values. And Section 6 closures with the conclusions and prospect research.

## 2. Background

In this section, a more detailed description of the existing anti-collision protocols is presented. Afterwards, bit-tracking technology and a bit-tracking protocol are presented.

### 2.1. Background

Various multi-access procedures have been developed in order to separate physically the transmitters' signals [3]. They are classified into Space Division Multiple Access (SDMA), Frequency Division Multiple Access (FDMA), Code Division Multiple Access (CDMA) and Time Division Multiple Access (TDMA):

— SDMA. Using a controlled directional antenna on the reader, it can point the beam at different zones to be read. However, these techniques are expensive and require complex antenna designs.
— FDMA. Transmission channel is split up into different carrier frequencies that are simultaneously available. It requires a complex receiver at the reader.
— CDMA. Tag IDs are multiplied with a pseudo-random sequence before transmission. It demands elevated power consumption.
— TDMA. Transmission channel is divided between the participants chronologically.

In RFID systems, TDMA procedures are the most used techniques in RFID and they have the largest group of anti-collision methods. These can be categorized in: Aloha-based protocols which are probabilistic, tree-based protocols which are deterministic, and hybrid protocols which are a mixture of the previous ones [6].

#### 2.1.1. Aloha-Based Protocols

The aloha protocol is the origin of the Aloha-based protocols. An improvement of that is the slotted-Aloha, which introduces the slot concept. A slot is a period of time during which the reader sends a command and the tags respond to the reader. Slotted-Aloha divides time into slots thus improving its throughput [3]. Later, framed-slotted-Aloha (FSA) is developed. In FSA all nodes must respond choosing a slot into a fixed length frame (a group of slots). As the throughput of the FSA decreases with the increase of the total amount of nodes, a dynamic-framed-slotted-Aloha (DFSA) is developed [7,8]. This protocol changes the length of the frame dynamically using an estimator to adjust the frame size. Some protocols like I-Code [8] change the frame size at the end of the last frame slot, and other algorithms, as the EPC C1G2 Slot Counter [9], adjust the frame size after a slot transmission. Early cited, the tag starvation problem affects probabilistic algorithms, this is a tag that may not be correctly read during a reading cycle. Besides, estimation involves some disadvantages [13]: an increase in the computational cost of the reader [8] and the tag [20]; an error that degrades the efficiency; and lastly, an initial frame length cannot be set according to the estimated number of tags.

#### 2.1.2. Tree-Based Protocols

The main feature of this kind of protocols is that they are deterministic. This is that all tags in the reader's interrogation zone are going to be identified. These protocols usually have simple design tags and work well with uniform set of tags but are slower than Aloha-based protocols. They can be categorized into [6]: Tree Splitting (TS), Query Tree (QT), Binary Search (BS) and Bitwise Arbitration (BTA).

**Figure 1.**
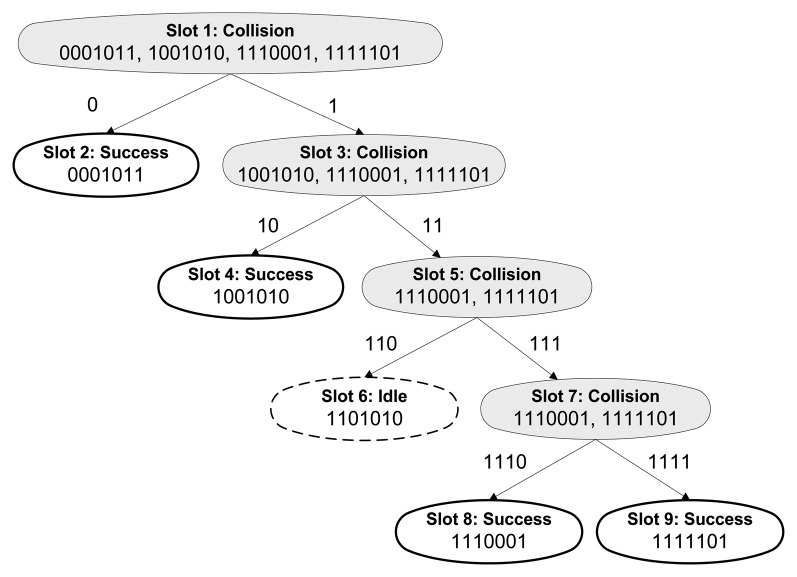
Example of the QT protocol.

A virtual tree to organize and identify each tag was firstly proposed by the authors of the TS in [10]. This algorithm splits the set of tags in *B* subsets (*B* > *1*) after a collision. These subsets become increasingly smaller until they contain one tag. The TS does not need clocking circuitry but they must maintain a counter, so if a tag get discharged, it loses cycle information. Moreover, the QT is proposed in [11]. The reader of the QT sends queries and tags, whose ID match that query, respond to the reader. After a collision, the reader increases the query with *1* or *0*, obtaining two new queries, and sending them repeatedly upon the successful response of all the tags. The process needs to go through all the possible queries to detect all the tags. QT is called memoryless because tags do not require any counter or memory. In Figure 1 an example of the QT protocol is shown. Additionally, the BS is another tree based protocol [21]. Tags compare their ID with a serial number sent by the reader. If the tag ID is equal to or lower than the serial number, the tag transmits its ID. Once a response is received at the reader, it decreases the serial number in case of a collision, or identifies the tag in case of a unique response. Lastly, the BTA protocols operate requesting tags to respond bit by bit. Tag responses must be synchronized in these protocols, so that identical responses could result in no collision. The Stack-based ID-Binary Tree algorithm (SIBT) [22] or the Bit Query (BQ) [23] use queries to cover a binary tree which height is the maximum tag ID.

#### 2.1.3. Hybrid Protocols

Tree- and Aloha-based protocols are combined in hybrid protocols to avoid their respective problems. There are mainly two combined protocols: one uses randomized divisions in tree-based algorithms, and the other one uses tree strategies after a collision in Aloha-based algorithms. The first kind of protocols such as Tree Slotted Aloha (TSA) [12] use a tree structure. Tag responses are sequenced in slots as in a FSA, and new frames are applied on collided tags. These kind of hybrid protocols require complex tags and carry the same problems as Aloha-based protocols, like the tag starvation. In contrast, in the second proposed protocols such as the Binary Tree Slotted Aloha (BTSA) [13], tags choose a slot randomly after a reader command. In case of a collision, a tree-based protocol is employed to identify tags. This variation of the hybrid protocols requires an initial estimation of the frame that determines the performance of the protocol.

### 2.2. Bit-Tracking Technology

Bit-tracking technology enables the reader to know which and where the collided bits in a collision slot are, using Manchester coding [3,17]. This codification defines the value of a bit as a voltage transition. A bit *‘0’* is coded by a positive transition and a bit *‘1’* by a negative transition. A collision occurs when two or more tags transmit different bits and they cancel each other out. Although a ‘no transition’ state is not allowed in Manchester coding, it can be used to trace the collision to an individual bit. Bit-tracking, therefore, requires all tags within the interrogation zone to transmit their data synchronously. That is a reasonable assumption since the propagation time is insignificant.

**Figure 2.**
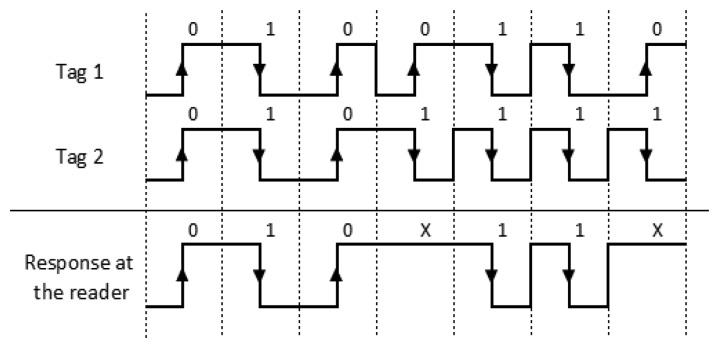
Example of Manchester coding.

Figure 2 shows an example of Manchester coding. Two tags are sending their IDs (*‘0100110’;‘0101111’*). The interfered signal received by the reader is *‘010X11X’*, where *X* represents a collision because there is no voltage transition during that bit time. This example shows collisions in the 4th and 7th bits. This information is used to identify tags faster and decrease the number of collisions in an identification round.

### 2.3. Collision Tree (CT)

The collision tree (CT) protocol [16,17] is a QT based protocol that implements bit-tracking. CT uses Manchester coding to seek the first collided bit so as to split the tags into two subsets. For a query *q_1_q_2_*…*q_L_* of length *L*, where *q_i_ ϵ {0,1}*, tags matching the reader query respond their remaining ID bits *p_1_p_2_*…*p_c_*…*p_T_*, of length *T*, where *p_i_ ϵ {0,1}* and *p_c_* is the first collided bit. The reader then, assembles two new queries *q_1_*…*q_L_ p_1_*…*p_c-1_‘0’*, which will match the first subset of tags and *q_1_*…*q_L_ p_1_*…*p_c-1_‘1’*, which will match the other subset.

**Figure 3.**
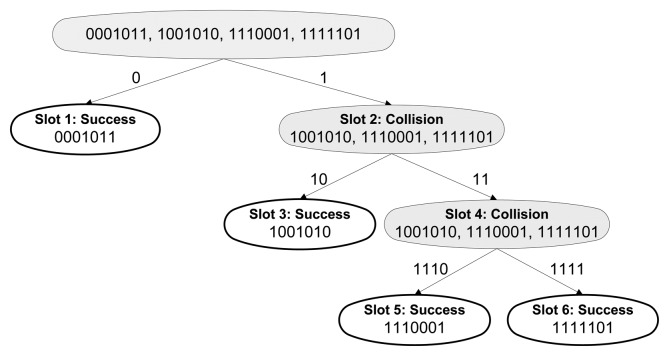
Example of CT protocol.

The CT protocol decreases the number of collisions compared to the QT and removes idle slots. All new queries are generated according to a collided bit, assuring that both new queries are going to be responded by at least two tags. Figure 3 shows an example of the identification of five tags using CT. The protocol eliminates idle slots and, extending the prefixes dynamically, outperforms QT with less slots and collisions.

## 3. Window Methodology

Aloha-based protocols are probabilistic and rely on more sophisticated tags than tree-based ones, which are deterministic [6]. However, the tags of the tree-based protocols usually need to transmit a higher number of bits to be identified. Because we aim to preserve low tag complexity we concentrate in tree-based protocols. Specifically, we have focused on tree-based protocols that use queries to identify the tags. Besides, we attempt to maintain the memoryless feature of QT based protocols to obtain a simple tag. In most tree-based protocols, matching tags respond with their full ID and there are lots of tag responses that end up colliding during an identification round. Therefore, there are a large number of tags whose responses are misunderstood by the reader, with the corresponding loss of time and energy.

### 3.1. Protocols Based on Queries

In the literature review there are two protocols based on queries: the QT protocol [11] and the SIBT protocol [22]. Each of them represents a strategy of tag identification:

— Large number of bits per slot: earlier mentioned, a QT slot is composed of a reader query and the matching tags responses of their IDs. The main advantage of this strategy is the complete identification of a tag in one slot. However, the loss of time and bits transmitted in a collision slot is highly remarkable.
— Small number of bits per slot: in the SIBT protocol, a slot consists on a reader query and one bit response from the tags. In a collided slot two ID bits are identified simultaneously. However, the increase in the number of bits of the tag ID causes an increase in latency.

### 3.2. The Window

**Figure 4.**
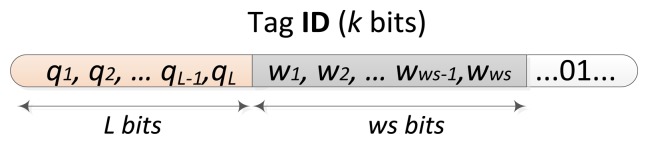
Structure of a tag ID.

The window methodology, presented in [18,19], restricts the number of bits transmitted by the tags. We call ‘window’ the bit-string a tag must transmit when it matches the query received from the reader. Tags that match the query received transmit synchronously the amount of bits specified by the window size parameter, *ws*, instead of their full ID with an attached cyclic redundancy check (CRC) string used to detect collisions. How the tag ID is organized is shown in Figure 4, where *k* is the length of the tag ID. A tag matching the query received from the reader, *q_1_,q_2_*…*q_L_*, transmits a bit-string of the following *ws* bits (window bits, *w_1_*,*w_2_*…*w_ws_*). The main idea of the window is that the tag to be identified needs to transmit fewer bits than a tag which transmit the complete ID. This idea is applied to the QT protocol and an analysis of the influence of the window on the QT protocol is performed to confirm the decrease on the number of bits transmitted by the tags. Indeed, the number of collisions and idles are also decreased. Due to the good results obtained in the number of bits transmitted by the tags and the number of slots, the QT protocol with a window size of 64 bits is included in the comparison with the proposed window later in Section 5.

The window manages to decrease the latency of the protocol (time to read out tag IDs) by reducing the number of bits transmitted on a collision. However, the number of collisions is still a problem that can be reduced. Moreover, the window methodology requires a synchronization of the tags messages, which is assumed but not specified. In this paper we propose to apply the window to the CT protocol in order to solve these problems.

## 4. Collision Tree (CT) Protocol with Window

We propose a novel protocol applying the window to CT which uses bit-tracking technology. Earlier mentioned, CT is a QT-based protocol which implements bit-tracking to eliminate idle slots and decrease the number of collisions. Bit-tracking technology uses Manchester codification to trace the collision to an individual bit, and then take advantage of the bits received previous to a collision. On the other hand, the window methodology decreases the number of bits transmitted by the tags, requiring synchronized responses.

### Bit-Tracking Technology Combined with the Window

**Figure 5.**
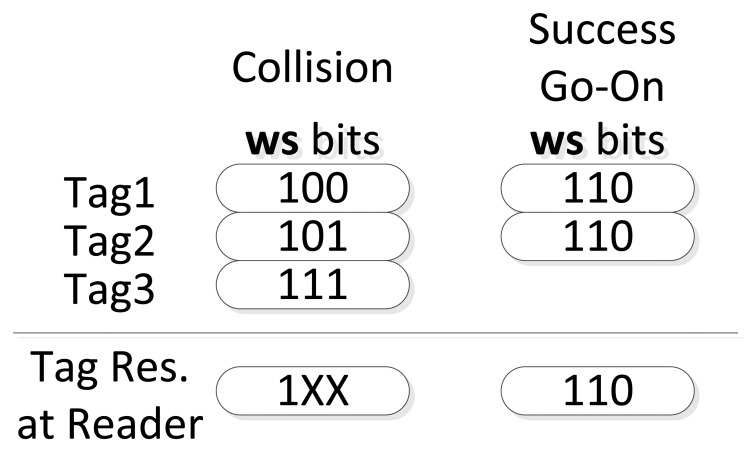
Slot statuses of the bit-tracking combined with the window (*ws* = 3).

The perfect matching of these strategies brings many benefits to the protocol both working independently as well as on concurrent operation. The combination also contributes to transform possible collisions into partial successes. However, the reader must interrogate tags until they transmit the last part of their ID. The main improvements produced by the combination of these technologies are:

— The window methodology demands simultaneous transmissions in order to detect the identical received bits as a unique response. The bit-tracking technology solves this problem thanks to the use of Manchester coding which facilitates synchronization.
— The window methodology also required a CRC to identify the type of slot. The bit-tracking allows the new window to avoid the use of these bit strings since the reader now is able to check for collided bits individually. This proceeding is shown in Figure 5.
— Idle slots are extinguished. The combination of both technologies provides an identification protocol without idle slots.
— Collision slots remain the same, but a new type of slot is produced. Go-On slots are partial success slots that are used to complete the tag ID, which is received in bit-strings of *ws* bits (window), see Figure 5.

The application of the window to CT is not only to reduce collisions in QT but also to decrease the number of bits transmitted by the tags to be identified. The adoption of the window methodology does not affect to the memoryless feature of the CT. Tags will transmit *ws* bits instead of the remaining bits *k-L* in response to a query. Therefore, the number of bits that a tag transmits in a collision slot decreases with respect to the non-windowed CT. That is the main idea of the window methodology.

Once the protocol begins, the reader transmits a query and three possible slot statuses can happen after a tag response:

— Collision slot: when various tags respond to a query and the responded windows are different. The reader is able to determine the collided bit.
— Go-On slot: when at least a tag responds its window and the reader is able to understand it. The received window is added to the last query transmitted as part of the received tag ID. However, if *L* + *ws* < *k*, a go-On slot is considered.
— Success slot: when a single tag responds its window bits and the reader completes the tag ID, *L* + *ws* = *k*.

**Figure 6.**
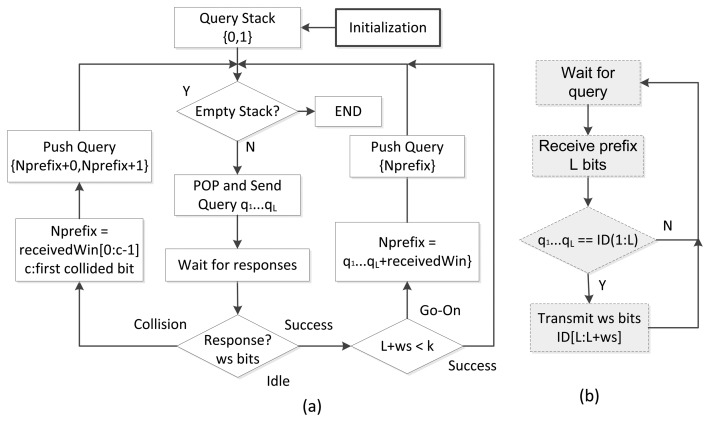
Flowchart of CT with window: (**a**) for reader, (**b**) for tags.

**Figure 7.**
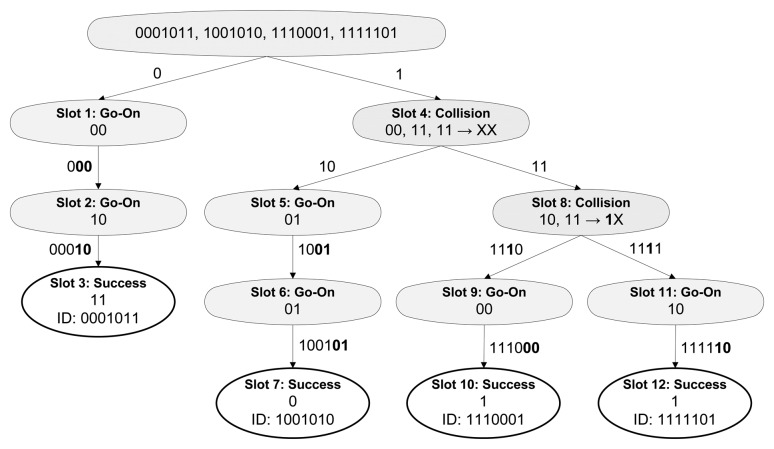
Example of CT with *ws = 2*.

A flowchart of the CT with window protocol is shown in Figure 6. An example of an identification round using CT with a 2 bit window size (*ws*) and 7 bit ID (*k*) tags is given in Figure 7. First, the reader (Figure 6a), initializes the procedure pushing two initial queries (*‘0’;‘1’*) into a Last input First output stack (LIFO) and then starts identification popping the first query from the stack and transmitting it to the tags. Whilst the reader stays waiting for the tag responses, tags receive the reader query (Figure 6b). Since one of the tags in the example matches the query received, it responds its following *ws* bits, *‘00’* (Figure 7). The reader, then, receives the tag response as a go-On slot, since a single tag responds in the proposed example and the received bits (*‘0’*+*‘00’*) are not enough to complete the tag ID, *L* + *ws* < *k*. The reader generates a new query and transmits it to the tags. Again, the same single tag responds and another go-On slot occurs. The procedure is repeated in close-loop until a successful slot, when the tag is undoubtedly identified and *L* + *ws* = *k*. After the success, a new query (*‘1’*) is popped from the stack and transmitted to the tags. In this case, three tags match the query and respond *ws* bits. The reader traces collisions in all the bits received, and therefore two new queries are obtained (*‘10’;‘11’*) (Figure 7). The next slots needed to identify the second tag are two go-Ons and a success, in the same as in the identification of the first tag. The next query (*‘11’*) is transmitted after the last success. A collision occurs again, but this time, the reader is able to trace the collision to the second bit, and two new queries are made with the query transmitted (*‘11’*), the understood part of the window (*‘1’*) and an *‘0*’ and *‘1’* to meet all the possibilities. As a result, the two new queries are *‘1110’* and *‘1111’*. Once they are transmitted, each branch of the tree needs an additional go-On slot to fully identify both tags. The reader checks again for the emptiness of the stack and then ends the procedure. The whole process is presented step-by-step in Table 1.

Undoubtedly, CT with the window performs using more slots than the non-windowed CT. However, in the presented example, Figure 7, the average number of bits transmitted by the tags in the CT with window is 6.75, while the CT tags need 11.25 bits.

## 5. Simulations

**Figure 8.**
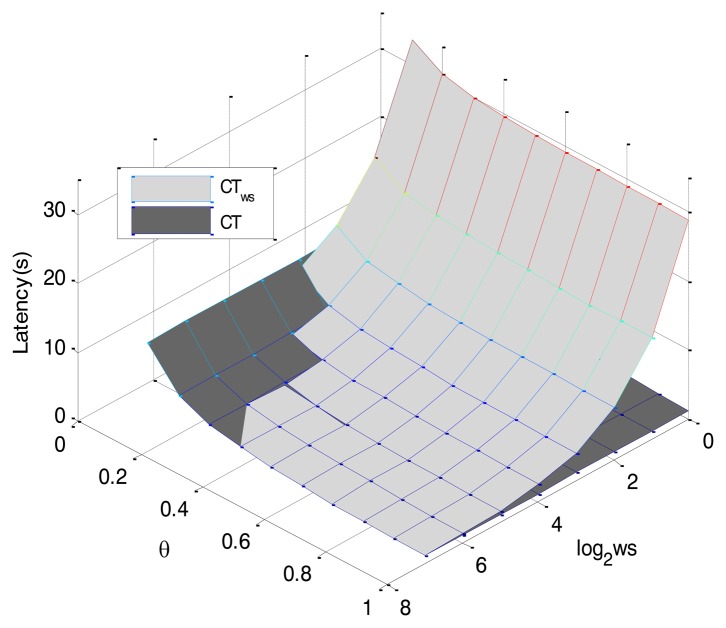
Latency as a function of *θ* and *ws*. The intersection between surfaces shows the benefits of the window in the CT protocol.

This section presents the simulation results of the CT protocol with the proposed window (CT_ws_) using Matlab R2012b with an evaluation of the outcomes. For the first simulation, a scenario with one reader and a group of 1,000 tags is proposed. The tags are uniformly distributed and *k* is assumed 96 bits. The tag IDs are dynamically generated for every simulation with varying random seed values. The simulated responses were averaged over 100 iterations for accuracy in the results. A comparison between the CT protocol [16,17] and the proposed CT_ws_ in terms of latency is presented. The time an RFID system spends on identifying the tags in the interrogation zone is known as latency. Many anti-collision protocols are verified using the number of slots, but not the slot duration (time), e.g., the time of a collided slot is longer than for an idle or a successful slot and is for this reason why we use the latency in this work. The simulation results are shown in Figure 8 as a function of *ws* and *θ*, where *θ* is given by Equation (1):

(1) $$\theta =\frac{\text{Tag data rate}}{\text{Reader data rate}}$$

First, *ws* variations are analyzed. CT_ws_ and CT performs similarly when *ws* = *k*. CT remains constant along the whole range of *ws* values since is not influenced by that parameter. Instead, the latency of CT_ws_ tends to increase as *ws* decreases. As it is deeply analysed in Section 5.2, low *ws* values require a higher number of slots, especially go-On slots to obtain the full ID of the tags. Besides, low *ws* values force the reader to send long queries (high number of bits), when it wants to obtain the last part of the tag ID. That is the main reason why low *ws* values cause the highest latency. However, tags transmit the least number of bits when low *ws* values are used

On the other hand, CT and CT_ws_ are influenced by *θ* parameter (Figure 8). Obviously, the lower the value of *θ*, the higher the latency of both protocols, because tags transmissions increase the total identification time. However, when *θ* values are below 0.5 and *ws* values are higher than *k/2*, CT_ws_ obtains better latency results than CT. The number of tag bits that CT_ws_ should transmit is considerably lower than the number of bits CT should transmit. That region indicated by the intersection between both protocol surfaces in Figure 8, shows the improvement of the CT_ws_ over the CT in latency.

**Figure 9.**
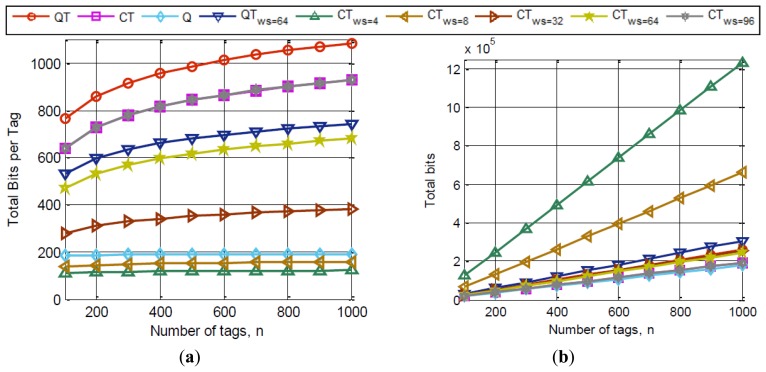
Tag performance of the CT_ws_ protocol compared to QT, QT_ws = 64_, Q and CT: (**a**) transmitted bits per tag; (**b**) total tag bits received at the reader.

Another simulation in a different scenario with a reader and a varying number of tags, *n*, from 100 to 1,000 is proposed. The tags are, again, uniformly distributed and *k = 96*. Results obtained are averaged over 100 iterations for accuracy in the results and the tag IDs have been dynamically generated for every simulation with varying random seed values. We consider the proposed CT_ws_ with different ws values and compare it to current anti-collision protocols: QT [11], the EPC C1G2 protocol (Q algorithm) [9], CT [16,17] and QT with a window size of 64 [19]. Figures 9a and b depict the results of this simulation that show the tags performance in the CT_ws_ protocol. The main idea of the proposed window is to manage the number of tags transmitted by the tags, to avoid transmitting a great number of bits in case of collision. It is clearly shown in Figure 9a that the smaller the window is, the fewer bits per tag that are transmitted. Moreover, results of the CT*_ws = k_* are identical to the results performed by CT. The QT protocol is outperformed by the QT with a window of 64 bits (QT*_ws = 64_*). And also, the CT with a window of the same size outperforms QT*_ws = 64_*. It should be noticed that the Q algorithm uses 16 bits random number tag responses (RN16) to reduce the number of bits transmitted by the tags, however, low window values outperform this protocol too. Thus, the benefits of the window are clearly stated. In addition, the combination of the bit-tracking and the window methodology works better due to the decrease of the number of collisions and idles with respect to the QT and the decrease in the number of tag bits transmitted.

Other results are obtained from the last exposed simulation. The total tag bits received at the reader are shown in Figure 9b. These are calculated as the addition of the number of bits received from the tags on every slot. When various tags respond their window bits to the reader, the total tag bits received is *_ws_*. And therefore, the total tag bits in a reading cycle are the number of slots multiplied by ws. CT*_ws = 96_*, which matches CT results and the Q algorithm show the best results on total bits transmitted. CT*_ws = 4_* suffers from a high number of bits transmitted by the reader, so it can be deduced that the higher the value of *ws*, the larger the number of bits transmitted by the reader. Figure 9b shows that the window applied to the bit-tracking protocol CT, produces better results than in QT, showing the improvement of combining bit-tracking and the window methodology.

### 5.1. Simulations under Fixed *θ* Values

**Figure 10.**
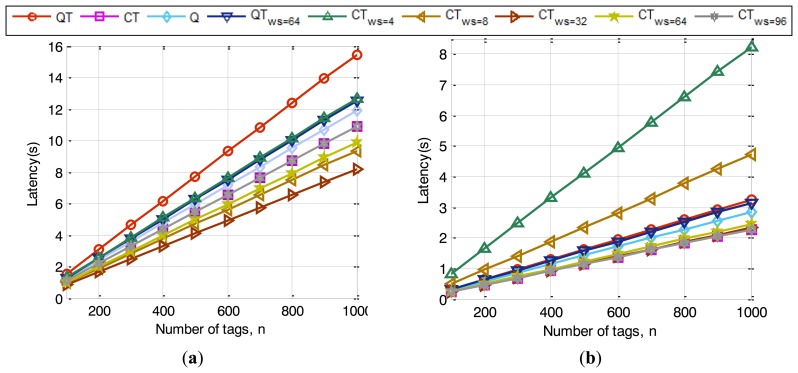
Simulated results of the system latency with various groups of tags compared to QT, QT_ws = 64_, Q and CT for two different *θ* values: (**a**) *θ = 0.1*; (**b**) *θ = 0.5*.

For the next simulation a similar scenario is proposed and compared to the same anti-collision protocols. Simulation results of the system latency as a function of *n*, for two values of *θ* (*0.1*; *0.5*) are shown respectively in Figures 10. Concluding remarks show the evidence of improved latency for the proposed CTws protocol when *ws* values are higher than 8 in Figure 9a with *θ = 0.1*. Especially the improvement is more remarkable in dense tag environments, where the likelihood of collision is higher. CTws presents a similar performance to CT when *ws = k*, and outperforms it ranging from *ws = 8* to *ws = 96*. QT*_ws = 64_* improves QT in latency, but it cannot outperform CT, because the bit-tracking technology provides no idle slots and a decrease of collisions which QT*_ws = 64_* cannot cope with. Also the Q algorithm, which uses RN16 tags responses, is outperformed by CT with window values from *ws > 8*. When *θ = 0.5*, latency results depicted in Figure 10b are not as promising as for *θ = 0.1*. Although the range from *32 ≤ ws ≤ 96* obtains similar results to the CT protocol, results shown in Figures 9 outperform CT in bits transmitted per tag. Thus, CT_ws_ provides simpler and less complex tags than the other compared protocols.

Summarizing, the window methodology provides a promising behavior in slow tags environments, where the reduction of bits transmitted by the tags is a critical issue to achieve low latency. The proposed CT protocol with the window is therefore a suitable candidate where low latency and low complexity are obtained.

### 5.2. Simulation Varying ws

**Figure 11.**
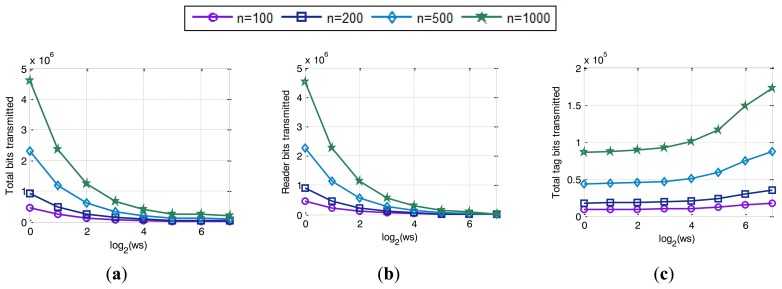
Simulated results of: (**a**) total bits transmitted; (**b**) reader bits transmitted and (**c**) total tag bits transmitted.

**Figure 12.**
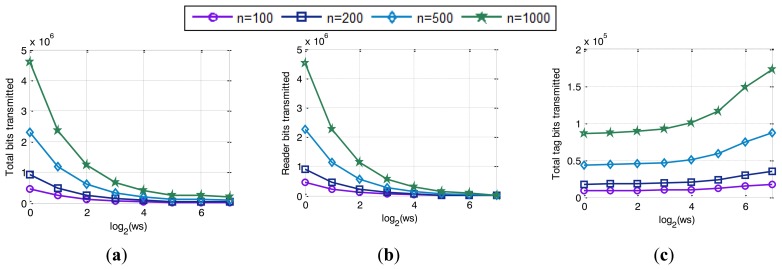
Simulated results of: (**a**) total bits transmitted; (**b**) reader bits transmitted and (**c**) total tag bits transmitted.

This simulation is planned as a function of *ws*, under different, *n*, groups of tags. The influence of the *ws* parameter on the QT_ws_ protocol is shown in Figures 11 and 12.

Simulated results in Figure 11a show the decrease of the total number of slots used in the identification process with the increase of *ws*. If a single tag matches a reader query, it transmits the number of bits specified by *ws*. Therefore the higher the value of *ws* is, the fewer slots that are required to obtain the full tag ID. On the contrary, a low *ws* value requires a high number of slots to identify a tag. In fact, the increase in the number of slots is caused by the need of go-On slots shown in Figure 11c, regardless of collision slots, Figure 11b, which remain constant. Besides, bit-tracking makes QT_ws_ to perform without idle slots and for that reason, the main increase of the number of slots is caused by the go-On slots. These slots are needed by the QT_ws_ to obtain the full ID of the tag being interrogated. However, go-On slots are critical to finish the identification cycle as soon as possible. Figure 11c shows that the smaller the *ws* is, the larger the number of go-On slots that are needed to identify all the tags in the interrogation zone. When tags are using low *ws* values, two related drawbacks cause the increase in the number of go-On slots: on the one hand, tags respond a few bits on each slot, which provides very low increases to the queries; and on the other hand, the reader needs many reader queries composed of a high number of bits to obtain the full ID of the tag. As a result, the reader needs higher number slots to identify all the tags in the interrogation zone. In addition, the increase in the number of go-On slots causes an increase in the total number of bits used, shown in Figure 12a, and specifically caused by the increased on the number of bits transmitted by the reader, Figure 12b. As it has been mentioned before, the reader transmits queries of a higher number of bits when tags transmit low *ws* values. Therefore, the number of bits transmitted by the reader increase, which also increases the total number of bits transmitted. Figure 12a shows the number of bits transmitted to identify all the tags in the interrogation zone. This value is calculated as the number of bits transmitted by the reader in a reading cycle, Figure 12b, plus the number of bits received from the tags at the reader, Figure 12c. These total number of bits decrease with the increase of *ws*. Although a small *ws* decreases the number of bits transmitted by a tag, Figure 12c, the improvement obtained is overwhelmed by the number of bits sent by the reader. However, when medium *ws* values are used, *θ* parameter determines the performance of the protocol in terms of latency due to the limitations in speed of the tags.

Summing up, the CT_ws_ protocol performs like the CT protocol when *ws* values are near *k*. In contrast, low *ws* values provide a great reduction of the bits transmitted by the tags, at the expense of an increase in the number of slots. A great number of go-On slots are required to accomplish the identification. That causes an increase in the total number of bits transmitted in a reading cycle. However, results obtained for medium *ws* values and low *θ* values show an improvement of the CT_ws_ in terms of latency. The decrease of the number of bits transmitted by the tags is reflected in a low latency identification round.

### 5.3. Selection of ws

At this point, a proper value of *ws* can be selected to face the identification process of a set of tags. A proper selection of *ws* should be made according to the specifications of the RFID system. Reader data rate and tag data rate should be taken into account. For that reason, when *θ* values are near 1, the improvement caused by low *ws* values in the number of bits transmitted by tags is overwhelmed by the need of the reader to transmit a higher number of bits. And in the end, latency does not offer good results. Thus, a high value of *ws* should be chosen. On the contrary, when low *θ* values are available, the best latency performance is obtained for medium *ws* values, around the range from 16 to 64. In this case, CT and QT are clearly outperformed by CT_ws_.

## 6. Conclusions

A new protocol combining bit-tracking technology and the window methodology is presented here. As both technologies are complementary, the window [18,19], which manages the number of bits transmitted by a tag, and the bit-tracking technology, which is used to extinguish idle slots and decrease collisions in the process of tag identification, are coordinated to solve the tag collision problem. The window has been applied to one of the most popular bit-tracking tree protocols, the CT, and has been compared under certain conditions. The resulting algorithm, keeps the memoryless feature of the CT. An analytical framework has been designed to compare the performance of both algorithms. Results obtained show that the bigger the *ws*, the more similar to the CT is the proposed CT_ws_ protocol. However, the number of bits transmitted per tag is reduced for all values of *ws*. Simulation results show that the CT_ws_ outperforms CT and QT when the values of *θ* are under the range of *0.4*.

In this paper, we propose to combine our previously developed window methodology, whose positive results were presented in [18,19] applied to the QT protocol, with the bit-tracking technology. In this manner, not only is the number of bits transmitted per tag decreased, but also the number of collisions. Thereby, the window methodology is applied to the collision tree (CT) protocol [17], which uses bit-tracking technology to recognize the first collided bit. The proposed window allows the CT protocol to perform in the same manner as a bit by bit algorithm for a small window size (*ws*) values or similarly to the CT for large *ws* values close to the ID length (*k*). We present a number of simulations to analyze the influence of the window in the CT protocol. Since the architecture of the RFID receivers, the encoding methods and the modulation used can determine tags data rate, and usually it is lower than the reader data rate [9], we have proposed a simulation considering reader and tags data rates, to conclude how the data rate affects the proposed window.

### Future Work

This work has been performed to obtain some conclusions of the performance of a tree based protocol using bit-tracking technology with the window methodology that controls the number of tag bits transmitted on each slot. A new anti-collision protocol based on the CT and the window methodology is going to be designed. Bearing in mind the conclusions obtained, a proposed protocol with dynamic window is expected to be designed. The window dynamic methodology will try to adapt the size of the window according to the conditions of the identification round. Therefore it will decrease the number of bits transmitted by the tags, specifically under collisions. That will bring an improvement in the total number of bits transmitted and will decrease the energy consumed by the RFID system. Moreover, the protocol will exploit correlated sets of tags decreasing the number of slots and improving the efficiency.

## Figures and Tables

**Table 1.** Example of an identification cycle of CT with *ws = 2*.

**Slot**	**Reader** **Query**	**Tag 1** ***0001011***	**Tag 2** ***1001010***	**Tag 3** ***1110001***	**Tag 4** ***1111101***	**Reader** **Interpretation**	**Slot Type**
1 -	*0*	*00*	-	-	-	*00*	Go-On
2 -	*0***00**	*10*	-	-	-	*10*	Go-On
3 -	*000***10**	*11*	-	-	-	*11*	Success–Tag 1
4 -	*1*	-	*00*	*11*	*11*	*XX*	Collision
5 -	*1***0**	-	*01*	-	-	*01*	Go-On
6 -	*10***01**	-	*01*	-	-	*01*	Go-On
7 -	*1001***01**	-	*0*	-	-	*0*	Success–Tag 2
8 -	*1***1**	-	-	*10*	*11*	*1X*	Collision
9 -	*11***10**	-	-	*00*	-	*00*	Go-On
10 -	*1110***00**	-	-	*1*	-	*1*	Success–Tag 3
11 -	*11***11**				*10*	*10*	Go-On
12 -	*1111***10**				*1*	*1*	Success–Tag 4
